# Effect of the Elaboration Method on Structural and Optical Properties of Zn_1.33_Ga_1.335_Sn_0.33_O_4_:0.5%Cr^3+^ Persistent Luminescent Nanomaterials

**DOI:** 10.3390/nano13152175

**Published:** 2023-07-26

**Authors:** Guanyu Cai, Luidgi Giordano, Cyrille Richard, Bruno Viana

**Affiliations:** 1 Chimie ParisTech, CNRS, Institut de Recherche de Chimie Paris (IRCP), Université PSL, 75005 Paris, France; guanyu.cai@chimieparistech.psl.eu (G.C.); luidgi.giordano@chimieparistech.psl.eu (L.G.); 2 Université Paris Cité, CNRS, INSERM, Unité de Technologies Chimiques et Biologiques pour la Santé (UTCBS), Faculté de Pharmacie, 75006 Paris, France

**Keywords:** persistent luminescence, phosphors, nanomaterials, Zn_1.33_Ga_1.335_Cr_0.005_Sn_0.33_O_4_, hydrothermal, solid state

## Abstract

Near-infrared (NIR) persistent luminescence (PersL) materials have demonstrated promising developments for applications in many advanced fields due to their unique optical properties. Both high-temperature solid-state (SS) or hydrothermal (HT) methods can successfully be used to prepare PersL materials. In this work, Zn_1.33_Ga_1.34_Sn_0.33_O_4_:0.5%Cr^3+^ (ZGSO:0.5%Cr^3+^), a newly proposed nanomaterial for bioimaging, was prepared using SS and HT methods. The results show the crystal structure, morphology and optical properties of the samples that were prepared using both methods. Briefly, the crystallite size of the ZGSO:0.5%Cr^3+^ prepared using the SS method is ~3 µm, and as expected, is larger than materials prepared using the HT method. However, the growth process used in the hydrothermal environment promotes the formation of ZGSO:0.5%Cr^3+^ with more uniform shapes and smaller sizes (less than 500 nm). Different diameter ranges of nanoparticles were obtained using HT and ball milling (BM) methods (ranging from 25–50 nm) and by using SS and BM methods (25–200 nm) as well. In addition, the SS-prepared microstructure material has stronger PersL than HT-prepared particles before they go through ball milling to create nanomaterials. On the contrary, after BM treatment, ZGSO:0.5%Cr^3+^ HT and BM NPs present higher PersL and photoluminescence (PL) properties than ZGSO:0.5%Cr^3+^ SS and BM NPs, even though both kinds of NPs present worse PersL and PL compared to the original particles before BM. To summarize: preparation methods, whether by SS or HT, with additional grinding as a second step, can have a significant impact on the morphological and luminescent features of ZGSO:0.5%Cr^3+^ PersL materials.

## 1. Introduction

Persistent luminescence (PersL) refers to the phenomenon where a material continues to emit light after the excitation source has been removed [1]. PersL is applied in many fields, such as anti-counterfeiting [2,3], information storage [4,5,6,7,8], thermal sensors [9], lighting [10,11,12,13,14,15] and imaging [16,17,18,19]. In terms of imaging, the development of nanoprobes that can emit deep-red or near-infrared (NIR) light has attracted the attention of the scientific community [20,21,22,23,24,25,26]. Specifically, NIR PersL is an emerging imaging modality that is quickly gaining popularity in the field of bioimaging, due to the key advantage of PersL being able to provide a long-term, low-background signal, making it ideal for sensitive imaging applications [27,28,29,30,31,32]. PersL can be easily activated with UV/visible light and X-rays, making it a versatile tool for imaging in a range of environments and conditions [33,34,35,36].

We first reported these persistent luminescence (PersL) Zn_(1+x)_Ga_(2−2x)_Sn_x_O_4_:Cr^3+^ spinel materials at micrometric size in 2020 [37], following our previous results obtained on ZnGa_2_O_4_:Cr^3+^ in 2011 [38]. Past works mainly focused on the Cr^3+^-doped gallate materials, such as ZnGa_2_O_4_: Cr^3+^(ZGO) [18,39,40,41,42,43], Zn_(1+x)_Ga_(2−2x)_Ge_x_O_4_: Cr^3+^(ZGGO) [44] and Zn_(1+x)_Ga_(2−2x)_Sn_x_O_4_:Cr^3+^(ZGSO) [37]. Furthermore, recent papers dealing with spinel materials doped with Ni, Yb and Er ions have recently been reported [3,8,24,45]. Important applications of PersL were documented for in vitro and in vivo imaging, and these applications were widely developed through the respective authors’ research laboratories [46,47,48]. Cr^3+^ is an ideal candidate that provides the deep-red/near-infrared PersL at ~700 nm due to the ^2^E → ^4^A_2_ transition, with some contribution of a large band in longer wavelengths from the ^4^T_2_ → ^4^A_2_ transition [49,50,51]. Through Sn^4+^-doping, the persistent luminescent performance of Cr^3+^-doped zinc gallate is improved due to the increase of spinel antisite defects, especially for visible-light charging [37], which makes it very promising for bioimaging applications. Some researchers have worked on different synthesis methods, however, there are no reports regarding the comparison of various synthesis methods within the same paper, namely the solid-state (SS) synthesis method with ball milling (BM) and hydrothermal synthesis (HT) method with BM. Furthermore, PersL is quite interesting to study as the property is very sensitive to the defects of crystalline quality. Indeed, traps and recombination centers should be carefully controlled. 

**Scheme 1:**
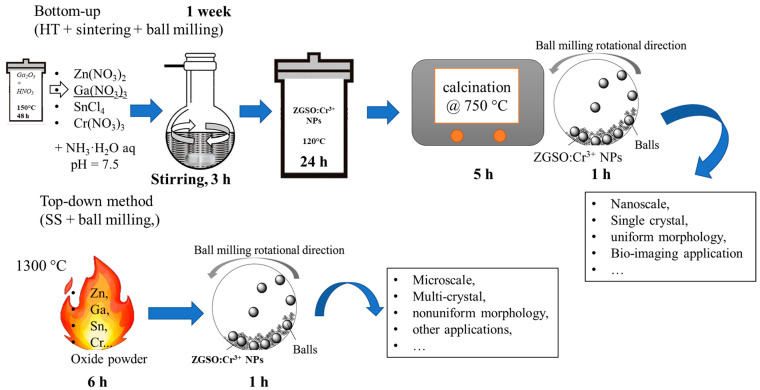
_33_Ga_1.335_Sn_0.33_O_4_:0.5%Cr^3+^ PersL phosphors, their properties and suggested applications.

In this study, we prepare Zn_1.33_Ga_1.335_Sn_0.33_O_4_:0.5%Cr^3+^ (ZGSO:0.5%Cr^3+^) PersL materials using both SS and HT methods, as shown in Scheme 1. Even though these two methods have been widely used for other materials in the industry, they have yet to be applied to ZGSO:0.5%Cr^3+^. We then report our comparison of the structure, morphology and optical properties, and use BM to downsize these compounds to nanoscale. Following this procedure, we provide details on the optical features of ZGSO:0.5%Cr^3+^ nanomaterials. Finally, in order to move toward applications, the properties of ZGSO:0.5%Cr^3+^ while suspended in water are presented, including concentration and size effects. This paper suggests that ZGSO:0.5%Cr^3+^ nanoparticles could be valuable probes for further bioimaging applications, using the appropriate synthesis method.

## 2. Materials and Methods

### 2.1. Materials

For the solid state method: zinc oxide (99.99%, Strem Chemicals, Newburyport, MA, USA), gallium oxide (99.99%, Strem Chemicals, Newburyport, MA, USA), tin oxide (99.99%, Strem Chemicals, Newburyport, MA, USA), chromium oxide (99.99%, Alfa Aesar, Karlsruhe, Germany).

For the hydrothermal method: zinc nitrate hexahydrate (>99%, Alfa Aesar, Karlsruhe, Germany), gallium oxide (99.999%, Alfa Aesar, Karlsruhe, Germany), nitric acid (35 wt%), tin chloride pentahydrate (98%, Alfa Aesar, Karlsruhe, Germany), chromium (III) nitrate nonahydrate (99.9%, Alfa Aesar, Karlsruhe, Germany), ammonia solution (30 wt%), hydrochloric acid (50 mmol L^−1^). All the chemicals were used as received without further purification.

### 2.2. Hydrothermal (HT) Method

The synthesis of ZGSO:0.5%Cr^3+^ refers to our previous reports [19,41], in which the tin concentration was optimized. In this method, 8.94 mmol of gallium oxide (99.999%, Alfa Aesar) was dissolved in 10 mL of concentrated nitric acid (35 wt%) under hydrothermal conditions for 48 h at 150 °C. Next, 10 mL of a solution containing 17.82 mmol of zinc nitrate hexahydrate (>99%, Fluka), 4.42 mmol tin chloride pentahydrate (98%, Alfa Aesar) and 0.07 mmol chromium (III) nitrate nonahydrate (99.9%, Alfa Aesar) was added to the vessel under vigorous stirring. All the chemicals are designed corresponding to the stoichiometric composition of Zn_1.33_Ga_1.335_Cr_0.005_Sn_0.33_O_4_ (ZGSO:0.5%Cr^3+^). The pH level of the solution was adjusted to 7.5 using ammonia solution (30 wt%), the mixture was stirred for 3 h at room temperature and then transferred to a stainless-steel autoclave, in which it was kept for 24 h at 120 °C. Under these conditions, the hydrothermal synthesis method ranges from about 10^5^–10^7^ Pa (1–100 bars) considering hydro and solvothermal methods, but at a temperature of about 120 °C the pressure does not exceed 40 bars [52,53]. The resulting solid was washed several times with water and ethanol and subsequently dried at 60 °C for 2 h. 1 g of the obtained solid was sintered in air at 750 °C for 5 h in a tubular oven.

### 2.3. Solid State (SS) Method

The ZGSO:0.5%Cr^3+^ powder sample was synthesized by a high-temperature solid-state method beginning with binary oxides. According to the stoichiometric ratio of the compounds, the raw material mixture was prepared. Briefly, 17.82 mmol of ZnO, 8.94 mmol of Ga_2_O_3_, 4.42 mmol of SnO_2_ and 0.035 mmol of Cr_2_O_3_, corresponding to the stoichiometric composition of Zn_1.33_Ga_1.335_Cr_0.005_Sn_0.33_O_4_ (ZGSO:0.5%Cr^3+^), were weighed and ground homogenously in an agate mortar and the mixture was introduced into an alumina crucible. Next, the crucible was placed in a high-temperature furnace at 1300 °C for 6 h in the air to produce the final sample [38]. After cooling to room temperature, the phosphors were ground into fine powders.

### 2.4. BM Treatment

The ZGSO:0.5%Cr^3+^ powders (~500 mg) prepared using both methods were crushed by a Pulverisette 7 Fritsch Planetary Ball Mill, with the addition of 1 mL of 5 mM HCl solution, at the speed of 1000 rpm for 1 h. The mixture was transferred into a round-bottom flask and vigorously stirred for 24 h at room temperature. The final ZGSO:0.5%Cr^3+^ NPs were selected from the polydisperse colloidal via centrifugation on a SANYO MSE Mistral 1000 (SpectraLab Scientific Inc. 38 McPherson St. Markham, ON, Canada) at 3500 rpm for 5 min. To optimize the ball milling conditions using the Pulverisette 7 Fritsch Planetary Ball Mill program, it is possible to fine tune the speed (between 0~1000 rpm) and time (0~4 h). By changing these parameters, we found the best conditions (at a speed of 1000 rpm for 1 h), as proposed in the manuscript.

### 2.5. Pellets Preparation for Spectroscopy Measurement

For all dry powder luminescence measurements via spectroscopy, a pellet containing 60 mg of the prepared material and 180 mg of KBr was prepared. We compacted the powder to form a whole pellet body with *ϕ*10 mm, with 2 mm thickness under uniaxial pressure of 5 MPa. Pellet samples are further used for optical spectroscopy measurements.

### 2.6. Characterization

#### 2.6.1. X-ray Diffraction (XRD)

XRD of ZGSO:0.5%Cr^3+^ was performed on an X-ray diffractometer (XPert PRO, PANalytical, Malvern Panalytical Ltd., Malvern, UK) equipped with a Ge111 single crystal monochromator selecting the K_α1_ radiation wavelength of the Cu X-ray tube (0.15405 nm).

#### 2.6.2. Transmission Electron Microscopy (TEM)

Observations of ZGSO:0.5%Cr^3+^ particles were carried out on a FEI^®^ Tecnai Spirit G2 TEM (ThermoFisher Scientific Inc., Hillsboro, OH, USA) working with an acceleration voltage of 120 kV. For the analysis, one drop of particle suspension is deposited on a carbon film-coated copper grid.

#### 2.6.3. Photoluminescence (PL)

Visible and deep red (or NIR-I) photoluminescence (PL) measurements of dry ZGSO:0.5%Cr^3+^ microstructured materials and NPs were performed using a CCD camera cooled at −65 °C and coupled to a monochromator (Acton Spectra Pro 2500, Princeton Instruments, Trenton, NJ, USA), at 300 grooves per mm, centered at 500 nm.

#### 2.6.4. PL Excitation Spectroscopy

PL Excitation measurements of the dry ZGSO:0.5%Cr^3+^ microstructured materials and NPs were carried out using an Agilent Cary Eclipse UV/Vis spectrophotometer.

#### 2.6.5. Persistent Luminescence (PersL)

The dry ZGSO:0.5%Cr^3+^ microstructured materials and NPs samples were thermally de-trapped at 70 °C before each experiment and then kept in the dark. The samples were excited with a UV lamp for 5 min at 290 K, and after cutting off the excitation, the afterglow was recorded for up to 60 min at the same temperature. The signal was followed with the same camera as in the PL experiment. Afterglow decay curves were obtained by integrating the intensity of the PersL spectra as a function of time.

#### 2.6.6. Photoluminescence Lifetime (PL Lifetime)

To measure the PL lifetime (PL decay curves), we use a pulse EKSPLA laser (8 ns, 10 Hz) with tunable wavelengths from UV to NIR for the excitation, and for the detection, an intensified CCD camera (ICCD) cooled at −65 °C, coupled to a monochromator (Acton Spectra Pro, Princeton Instruments), with a grating of 300 grooves per mm, centered at 500 nm. The data of the decay curve were collected using Winspec software V2.6A for some PersL decay profiles we used either the Imax Roper Pixis camera with an integration time of 1 s (and measured the decay just after stopping the excitation) or the Optima Biospace Lab camera described below.

#### 2.6.7. Thermoluminescence (TL)

Thermoluminescence (TL) measurements were performed using a closed-cycle He-flow cryostat (Sumitomo Cryogenics HC-4E) attached to a Lakeshore 340 temperature controller. The samples were cooled down to 10 K and irradiated with a UV lamp for 10 min. Next, the TL curves were recorded at a 10 K/min incline while heating up to 470 K. The signal was recorded using an ICCD camera (Roper Pixis 100) coupled to a visible monochromator (Acton Spectra Pro, Princeton Instruments), at 300 grooves per mm, centered at 500 nm).

#### 2.6.8. Persistence Luminescence of ZGSO:0.5%Cr^3+^ NPs Suspension

Dry ZGSO:0.5%Cr^3+^ NPs were weighted and dispersed into DI water filling 2 mL Eppendorf, (weighing, according to the targeted concentration, from 0.2 mg/mL to 2 mg/mL), via ultrasonic dispersion. ZGSO:0.5%Cr^3+^ NPs in suspension (series of concentration from 0.2 to 2 mg/mL) are transferred into a 96-well plate (0.3 mL for each suspension hole), and irradiated under a UV lamp for 2 min. The excited suspension was then observed using an Optima camera (Biospace Lab, Nesles-la-Vallée, France) for 5 min of acquisition in bioluminescence mode. After collection, M3-vision software was employed for data treatment.

## 3. Results and Discussion

### 3.1. Crystal Structure

**Figure 1:**
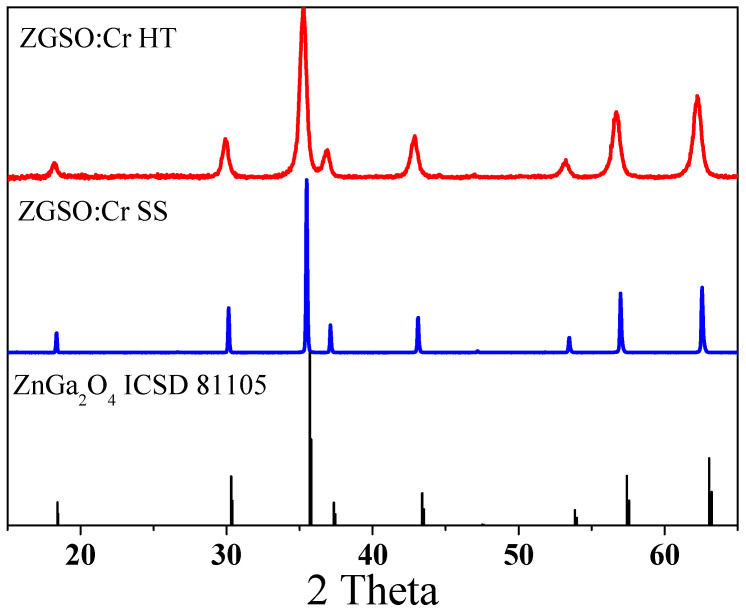
XRD patterns of Zn_1.33_Ga_1.335_Sn_0.33_O_4_:0.5%Cr^3+^ (ZGSO:0.5%Cr^3+^) prepared using solid-state (SS) or hydrothermal (HT) methods.

ZGSO:0.5%Cr^3+^ possesses a normal spinel structure belonging to the cubic lattice system with two tetrahedral and four octahedral sites per formula unit, as shown in Figure S2 in the Supplementary Materials. The ZGSO matrix is comprised of cubic, close-packed oxides with space group *Fd-3m* [37]. Ga^3+^ cation size (0.76 Å, CN = VI) is smaller than the Zn^2+^ (0.88 Å, CN = VI) and Sn^4+^ (0.83 Å, CN = VI) cations, respectively. The radius of trivalent cations in octahedral site Cr^3+^ (0.755 Å) is close to that of Ga^3+^, and therefore Cr^3+^ is inclined to occupy the octahedral Ga^3+^ sites. In this structure, a part of the Zn occupies the tetrahedral sites, while the rest of the Zn^2+^, as well as all Ga^3+^, and Sn^4+^ are expected to occupy the octahedral sites [41]. To confirm the crystal structure of our materials, Powder XRD measurements were conducted and analyzed, as shown in Figure 1. The XRD results show that ZGSO:0.5%Cr^3+^ can be successfully prepared using both HT and SS methods. All of the recorded diffraction peaks in the XRD pattern are basically consistent with the standard ZnGa_2_O_4_ card (ICSD No. 81105) with a slight shift in the diffraction peaks due to the presence of Sn^4+^ inside the structure with a larger radius than Ga^3+^.

### 3.2. Microscopic Investigation

**Figure 2:**
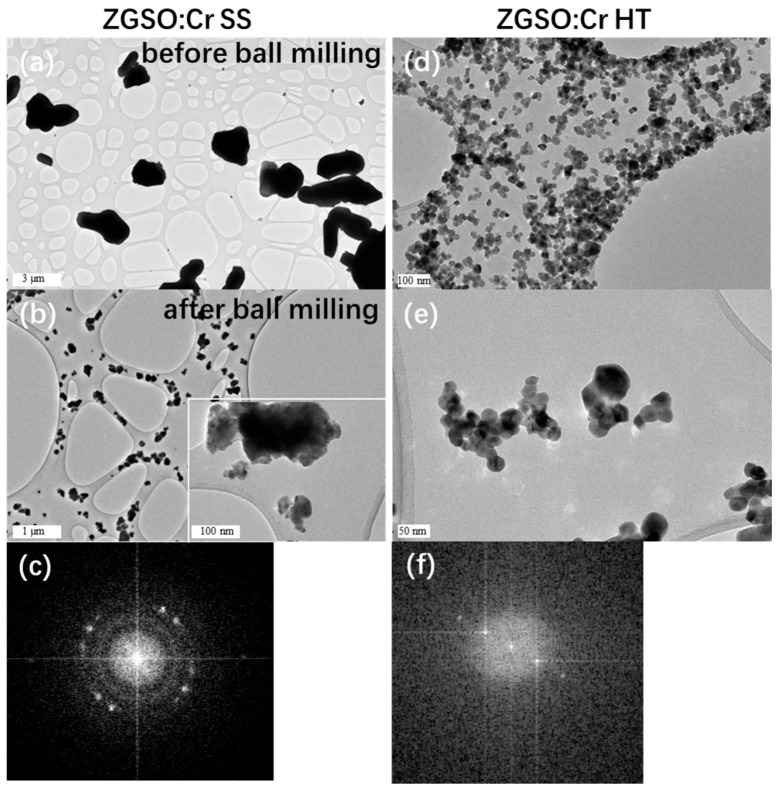
TEM images of ZGSO:0.5%Cr^3+^ (**a**) before and (**b**) after a BM treatment after SS method preparation, insert of (**b**) is part enlargement. (**c**) Electron diffraction (ED) of the ZGSO:0.5%Cr^3+^ SS. (**d**,**e**) TEM images of ZGSO:0.5%Cr^3+^ prepared by HT method after BM treatment. (**f**) Electron diffraction (ED) of the ZGSO:0.5%Cr^3+^ HT phosphors after BM, respectively.

Transmission electron microscopy (TEM) was used to determine the microstructure of the ZGSO:0.5%Cr^3+^ samples (see Figure 2), prepared using both methods, ZGSO:0.5%Cr^3+^ SS (a–c) and ZGSO: Cr^3+^ HT (d–f). These microscopy images show that particle sizes of ZGSO:0.5%Cr^3+^ prepared using the solid-state method are larger than 1 μm. In fact, most of the particles are about 3 μm (see Figure 2a). Their irregular shape is due to a higher degree of agglomeration, and the formation of crystalline structures, as in the electron diffraction (ED) results of the polycrystal, could be approximated by an annular diffraction spot (in Figure 2c). On the contrary, the growth process used in the hydrothermal environment promotes the formation of ZGSO:0.5%Cr^3+^ HT materials with smaller sizes (less than 500 nm, not shown here), and more uniform shapes (in Figure 2d) when compared to the ones obtained using the SS method. The ED of the particles prepared using the HT method also shows the formation of single crystal-like nanostructures with regular shapes (in Figure 2f).

To obtain nanoparticles from both methods, it is necessary to grind the particles a second time, to reduce the size of the grains. This leads to size dispersion, as seen in Figure 2b. Nanoparticles with different diameter ranges could be further extracted using SS and BM (sizes in the range of 25–200 nm) as seen in Figure 2b, and ranging from 25–50 nm for HT and BM, as seen in Figure 2d,e. Monodisperse and smaller average-sized nanoparticles are obtained for ZGSO:0.5%Cr^3+^ HT and BM in comparison to the ones prepared using solid-state and ball milling. Particles are selected via high-speed centrifugation (speed of 1000 rpm for 1 h). For example, ZGSO:0.5%Cr^3+^ NPs with average sizes of ~25, ~35 and ~50 nm can be separately selected for further measurements in Section 3.4. Usually, there is less than a 10% margin of error in determining the NPs sizes (for example, NPs diameter = 25 nm ± 2 nm; 50 nm ± 5 nm).

### 3.3. Optical Properties

**Figure 3:**
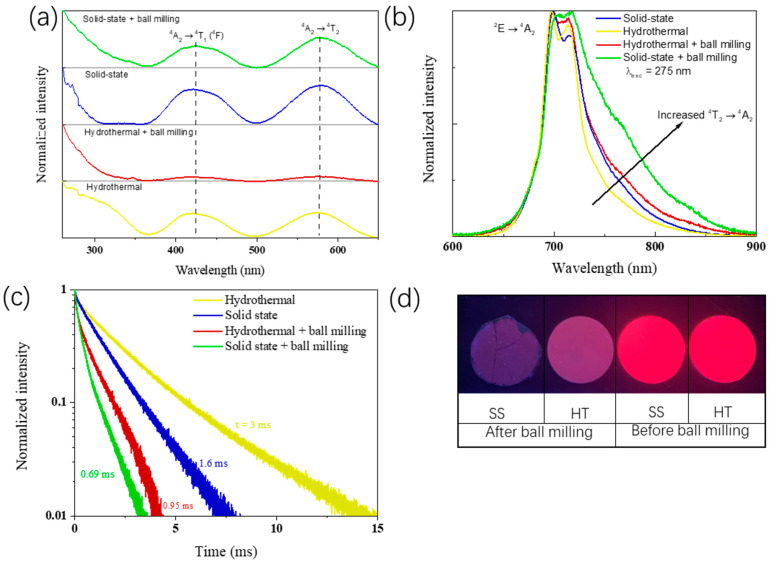
(**a**) PL Excitation with λ_em_ = 700 nm, (**b**) emission spectra with λ_exc_ = 275 nm, (**c**) lifetime curve (λ_exc_ = 450 nm), (**d**) PL digital images of the ZGSO:0.5%Cr^3+^ phosphors prepared using solid-state or hydrothermal methods, before and after BM treatment, in dry powder form during excitation with a UV lamp (λ_exc_ = 254 nm, room temperature).

The effect of the preparation method on the optical properties of the Zn_1.33_Ga_1.335_Sn_0.33_O_4_:0.5%Cr^3+^ (ZGSO:0.5%Cr^3+^) was also analyzed. Normalized PL excitation spectra monitoring emission at 700 nm (shown in Figure 3a) presents large UV excitation peaks (at wavelengths below 370 nm) for all samples. This can be attributed to the band gap energy of these materials. The other two excitation peaks located at ~425 nm and ~580 nm, respectively, typical of the trivalent chromium (^4^A_2_→^4^T_1_ and ^4^A_2_→^4^T_2_ transitions, respectively) can be observed as well. Throughout both synthesis methods and comparisons of excitation bands before and after BM, no band shift could be observed. This suggests that, in all cases, Cr^3+^ is under the same crystal field and environment. This indicates that Sn^4+^ content remains at the same level in both cases, as the introduction of Sn^4+^ instead of Ga^3+^ in this ZGSO matrix drastically modifies the crystal field around Cr^3+^, and therefore its excitation wavelength, as reported by Pan et al. [37]. The presence of these bands also indicates that Cr^3+^ can be directly excited through the ^4^A_2_ → ^4^T_1_ (^4^F) and ^4^A_2_ → ^4^T_2_ (^4^F) transitions. After BM treatment, the excitation peaks of both ZGSO:0.5%Cr^3+^ samples do not move, which means that the decrease in size to nanoscale does not change the Cr^3+^ crystal field.

As can be seen from the emission spectra obtained under excitation at 275 nm (UV excitation) in Figure 3b, both synthesized samples before BM have a strong emission peak at ~700 nm in the deep red (or NIR-I) range. While the emission peak is mainly caused by the ^2^E → ^4^A_2_ transition of Cr^3+^, there is a small contribution from the ^4^T_2_ → ^4^A_2_ transition, as these levels are in thermal equilibrium [38]. In relation to their emission bands, there is a higher PL intensity ratio of Cr^3+ 4^T_2_ → ^4^A_2_ transition over ^2^E → ^4^A_2_ transition in the solid-state samples compared to the samples obtained using the HT method [54,55]. When samples were treated by BM for HT and SS, an increased contribution of the ^4^T_2_ →^4^A_2_ transition was found. The increase of ^4^T_2_ → ^4^A_2_ to ^2^E → ^4^A_2_ ratio after BM suggests the creation of new non-radiative energy loss paths after obtaining the nanoparticles, possibly due to the formation of surface defects, which is a side effect of the BM procedure and also explains the poor signal to noise ratio in samples after BM. In Figure 3d, the image obtained during UV excitation of the samples clearly shows that after BM there is a significant decrease in the PL intensity.

**Figure 4:**
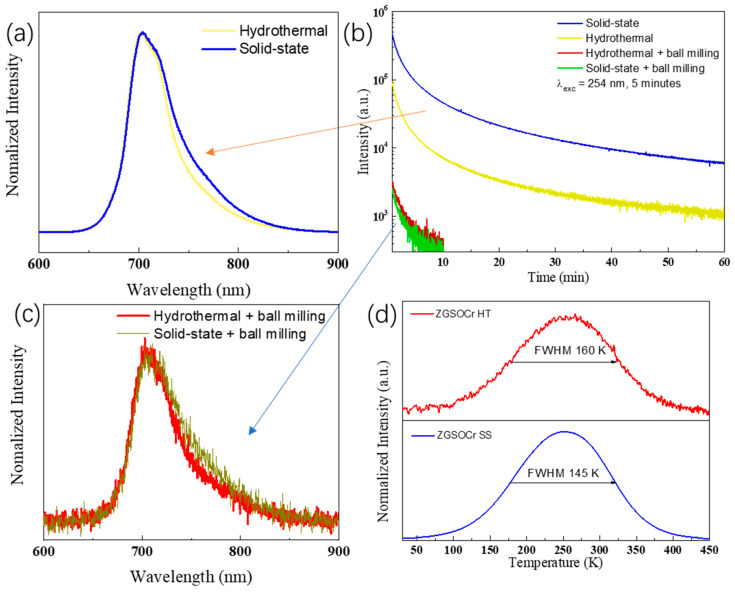
(**a**,**c**) PersL glow curves 5 s after ceasing UV excitation and (**b**) decay curves of ZGSO:0.5%Cr^3+^ phosphors prepared using solid-state or hydrothermal methods, before and after BM treatment. (**d**) thermoluminescence (TL) spectra of microscale ZGSO:0.5%Cr^3+^ phosphors prepared using solid-state or hydrothermal methods. All collections of PersL glow curves are started 5 s after stopping the 5 min UV excitation.

Lifetime was used for the evaluation of the optical properties of materials, with a focus on their efficiencies. For the ^2^E emission level of Cr^3+^ in ZGSO, a lifetime value of around 3 ms was found for the material prepared using the HT method, while for the material prepared using the SS method, its lifetime value is around 1.6 ms when excited at 450 nm. Even after changing the excitation wavelengths through a large excitation energy range from 254 nm to 550 nm, Cr^3+ 2^E manifold lifetime does not change significantly (Figure S1). It is at first quite surprising to find higher lifetime values for the material prepared using the HT method, as the calcination temperature is lower, which usually leads to lower crystallinity and the existence of additional non-radiative paths, decreasing the average lifetime. However, in PersL phosphors, as trapping is the most relevant effect, this suggests that during excitation, traps are being filled, which would reduce the lifetime of the manifold. This will be investigated in more detail in the following section (see Figure 4d) with thermoluminescence glow curves. In the case of ZGSO:0.5%Cr^3+^, one must also consider that in the solid-state elaboration method, there is also a higher contribution of the ^4^T_2_ → ^4^A_2_ transition, which also explains the lower lifetime value when compared to the samples prepared using the HT method. After BM, lifetime values for both preparation methods decrease. ^2^E manifold lifetimes decrease from 3 ms to 0.95 ms for hydrothermal method and from 1.69 ms to 0.69 ms for solid-state one, respectively, corroborating the BM effect on the PersL intensity. As for the PL intensity, lifetime measurements show that the BM creates alternative, non-radiative pathways, as the ^2^E manifold’s lifetime decreases drastically following the formation of nanoparticles.

To evaluate the PersL properties of ZGSO:0.5%Cr^3+^ prepared using solid-state (SS) or hydrothermal (HT) methods, before and after BM treatment, their decay curves in a longer time range were collected (in Figure 4b) by monitoring Cr^3+^ emission at ~700 nm after 5 min of UV excitation. The nature of the antisite defects and mechanism of persistent luminescence for this family of materials is well known [36,37] and will not be discussed in detail in this work. When comparing the PersL of the four samples, samples prepared using the solid-state method appear to be the most effective. When comparing both materials before BM, the higher synthesis temperature of the material prepared using the solid-state method is responsible for a higher crystallinity, and due to the thermodynamic nature of the defect formation, there is also a higher concentration of controlled defects useful for PersL using this synthesis method. Controlled defects mean that the defect’s depth is well adapted to the persistent luminescence, namely at about 0.5–0.7 eV below the conduction band. However, after BM and nanoparticles extraction, both materials have lost significant performance, with the one prepared using the HT method remaining slightly superior. The crucial effects of BM leading to performance losses can be attributed to the formation of quenching defects and non-radiative pathways, interfering with energy storage in controlled defects and antisites, for instance, favorable to PersL, thus decreasing their PersL performance [56]. In terms of emission profiles (see Figure 4a,c), the samples prepared by the solid-state method have a higher relative contribution of the ^4^T_2_ → ^4^A_2_ emission, as also recognized from photoluminescence features.

As PersL is closely related to the existence of traps, the traps depth profile was studied by their thermoluminescence glow curves (λ_exc_ = 254 nm, Figure 4d). Notice that traps depths about 0.5–0.7 eV below the conduction band roughly correspond to peaks in the thermoluminescence glow curves around 250 to 350 K. For both materials prior to BM, the thermoluminescence glow curve shows maximum emission around 255 K. This, as shown previously for ZGSO [19,37], suggests that an important part of ZGSO’s traps are outside the optimal temperature range for long persistent phosphors, being quickly thermalized at room temperature. However, this is indeed favorable for bio-imaging, as the intensity in the first minutes will therefore be enhanced [37]. In both cases, the bands are large, with full width at half maximum (FWHM) of 145 K and 160 K for solid state and hydrothermal methods, respectively. The broadness of this band is a consequence of the formation of Sn_Ga_° and Zn_Ga_’ defects acting as electron and hole traps, respectively, when Sn^4+^ is added to ZnGa_2_O_4_ [37]. In terms of absolute intensity, through signal-to-noise ratio comparison, the sample prepared by solid-state has a much higher thermoluminescence intensity. This is in correlation with the higher PersL of this sample, and is likely related, as stated previously, to the thermodynamics of defect formation at higher temperatures, allowing more energy storage.

### 3.4. Colloidal Stability and PersL Properties of NPs in Suspension

**Figure 5:**
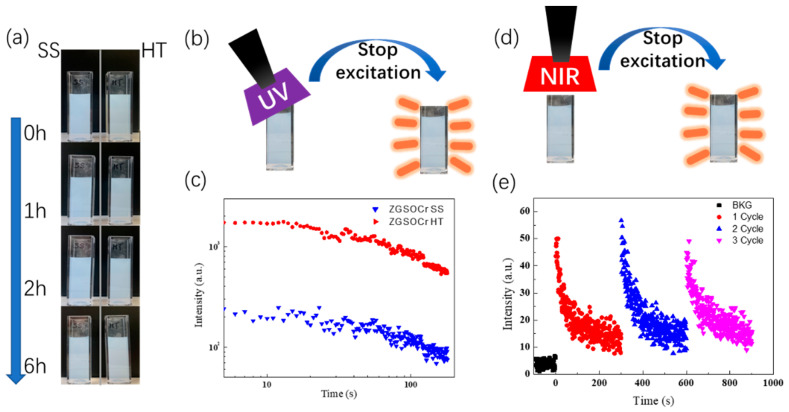
(**a**) Suspension stability measurement of the NPs obtained using the solid-state or hydrothermal methods, ZGSO:0.5%Cr^3+^ NPs dispersed in water for up to 6 h, (**b**) scheme of UV excitation for PersL of ZGSO:0.5%Cr^3+^ NPs suspension, (**c**) PersL decay curves of the suspension (0.2 mg/mL dispersed ZGSO:0.5%Cr^3+^NPs prepared using both methods). (**d**) scheme and (**e**) decay curve of NIR optical stimulated luminescence of the ZGSO:0.5%Cr^3+^ HT NPs with ~25 nm diameter in 0.2 mg/mL water suspension. (Excitation by 980 nm laser diode with three cycles).

Taking into account further applications of ZGSO:0.5%Cr^3+^ PersL NPs for bioimaging as presented previously [57], circulating NPs could have a long journey via blood circulation (pH ≈ 7.4) until they reach their target or are trapped by the liver, for instance. Therefore, suspension in water is an important step for the stability characteristic of the ZGSO:0.5%Cr^3+^ PersL NPs, even though suspension in water is different from the real in vivo system and environment. Respectively, ZGSO:0.5%Cr^3+^ SS and BM NPs and ZGSO:0.5%Cr^3+^ HT and BM NPs are washed via DI water twice and then dispersed into DI water via ultrasonic dispersion into. Both kinds of NP’s suspension samples with a concentration ~0.2 mg/mL are pipetted into a cave (3 mL). The stability of the NP’s suspension is characterized by the sedimentation behavior of NPs in water suspension. Figure 5a shows the colloidal stability of ZGSO:0.5%Cr^3+^ NPs dispersed in water after BM treatment of materials prepared using both the solid-state (SS) and hydrothermal (HT) methods. As can be seen in Figure 5a, up to at least 6 h, there are no apparent changes in the stability or decantation of the solid. Subsequently, the PersL NPs in suspension were excited by UV (254 nm) for 2 min, as shown in Figure 5b. The PersL decay curves of the suspension samples are reported in Figure 5c. PersL NPs in suspension with ZGSO:0.5%Cr^3+^ prepared using the HT method present higher intensity and longer durations than those prepared using the SS method, which is correlated to the PersL results obtained from the dry NPs after BM. This may be related to the controlled growth conditions and the smaller particles formed during the HT synthesis (as seen in TEM) and slower reaction rates in the hydrothermal environment. The HT reaction condition can help preserve the luminescent centers and reduce the formation of quenching defects even after BM. Thus, ZGSO:0.5%Cr^3+^ NPs can present more effective optical properties when made using the HT method. Furthermore, ZGSO:0.5%Cr^3+^ HT and BM NPs also show remarkable optical stimulated luminescence (OSL) properties (Figure 5d) when excited by a NIR laser diode. This corresponds to light-induced trap redistribution. The suspension of ZGSO:0.5%Cr^3+^ HT and BM NPs presents desirable optical stimulated PersL property over several cycles after excitation under 980 nm laser (see Figure 5e).

**Figure 6:**
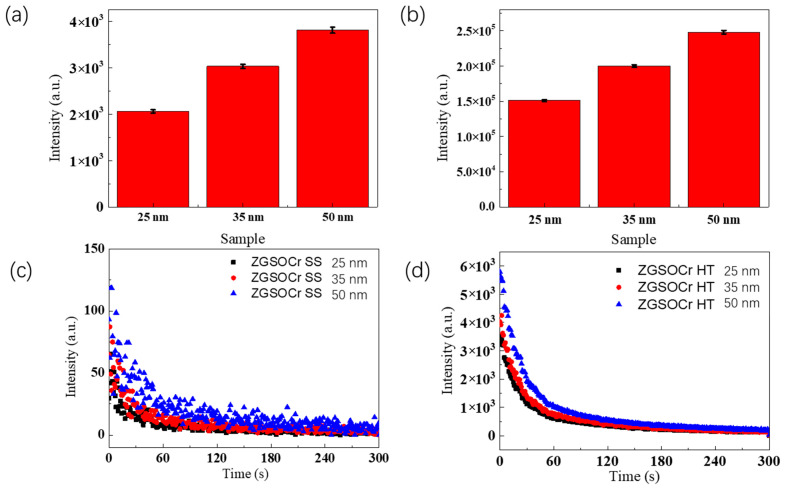
(**a**,**b**) Total counts of PersL of ZGSO:0.5%Cr^3+^ NPs prepared using solid-state and hydrothermal methods, with different diameters in suspension (in water) with 0.2 mg/mL and (**c**,**d**) their respective decay curves (acquisition time of 5 min, starting 1 min after stopping 254 nm excitation).

To better understand the nanoparticles’ size effect on PersL properties in suspension, suspension samples of ZGSO:0.5%Cr^3+^ NPs (0.2 mg/mL in DI water) were prepared, for both the SS and HT methods, using NPs with average sizes of 25, 35 and 50 nm (with <10% size errors, when determining average particle size under TEM). NPs were selected via careful centrifugation. Figure 6a shows the total counts of PersL intensity (5 min acquisition time), monitored at 700 nm, for the three ZGSO:0.5%Cr^3+^ NP sizes obtained using the SS method after BM treatment. Meanwhile, their decay curves are shown in Figure 6c. The same was repeated for ZGSO:0.5%Cr^3+^ NPs obtained using the HT method after BM, as can be seen in Figure 6b,d. All signal acquisitions of the suspension samples started 1 min after a 2 min irradiation with UV light. Higher average particle sizes yield higher persistent luminescence signal intensity, as they have a lower surface-to-volume ratio. Due to this lower ratio, less energy is lost through surface ligands and defects. When comparing both methods, one can observe that the PersL intensity of nanoparticles prepared using the HT method is about 50 times more intense than the one prepared using the SS method with similar particle sizes.

**Figure 7:**
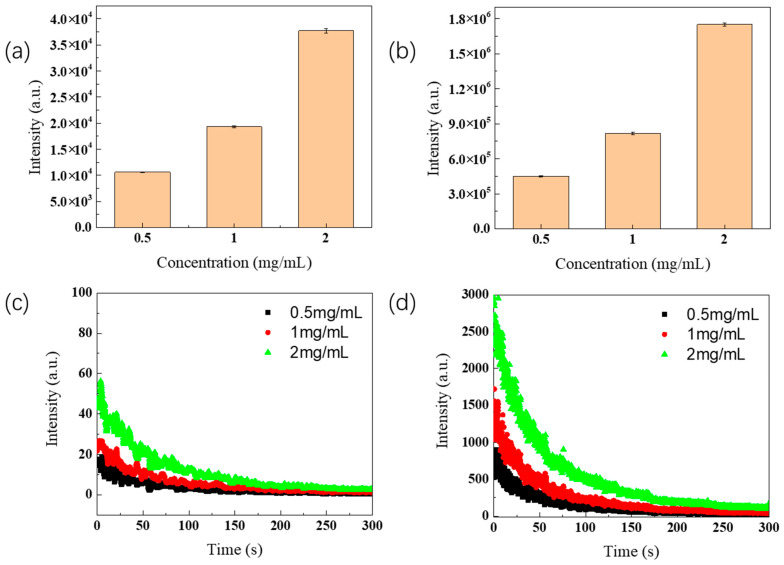
(**a**,**b**) Total counts of PersL decay of ZGSO:0.5%Cr^3+^ NPs prepared using solid-state or hydrothermal methods (~25 nm average diameter determined by TEM) with different concentrations in water suspension and (**c**,**d**) their decay curves profiles within 5 min, starting 1 min after the end of the 254 nm of UV excitation.

To understand the relationship between the PersL and the ZGSO:0.5%Cr^3+^NPs’ mass concentration in suspension, the suspension samples with a series of concentration (0.5 mg/mL, 1 mg/mL and 2 mg/mL) were prepared for both SS and HT methods, using nanoparticles of average size (~25 nm, as measured by TEM). Figure 7a shows the total counts of PersL (5 min. acquisition) monitored at 700 nm NPs obtained using the SS method, after BM treatment. Their respective decay curves are presented in Figure 7c. Similarly, the total counts of the ZGSO:0.5%Cr^3+^ suspensions prepared using the HT method and their respective decay curves are shown in Figure 7b,d. As expected, a concentration increase is responsible for a significant increase in collected PersL signals. The signal intensity collected from ZGSO:0.5%Cr^3+^ NPs obtained using the HT method is around 50 times higher than that of the NPs obtained using the SS method for a given concentration.

## 4. Conclusions

In this work, Zn_1.33_Ga_1.335_Sn_0.33_O_4_:0.5%Cr^3+^ (ZGSO:0.5%Cr^3+^) deep-red persistent luminescence phosphors were successfully prepared using solid-state (SS) and hydrothermal (HT) methods. The characterization results clearly show the significant impacts of the grinding method on the structural, morphological and luminescent properties of ZGSO:0.5%Cr^3+^. Briefly, the SS method, without further grinding, leads to irregularly shaped particles, and a crystallite size of the ZGSO:0.5%Cr^3+^ phosphor over 1 μm. Furthermore, SS-prepared micrometric phosphors exhibit high efficiency in terms of persistent luminescence (PersL) duration, which is correlated to the high-temperature reaction. This method leads to the narrowing of defects’ energy measured by thermoluminescence (TL) and their localization below the conduction band, as evidenced by the TL glow curve observed between 250–300 K. Even after mechanical ball milling (BM) treatment, the size of the NPs is hardly reduced to reach 25 nm, while their PersL intensity drastically decreases.

On the other hand, for those prepared using the HT method, the growth process of the nanoparticles in the hydrothermal environment promotes the formation of ZGSO:0.5%Cr^3+^ with more uniform shapes and smaller sizes, thus even after a BM treatment, the size of NP products are easily reduced, and their PersL is proportionally less reduced than NPs obtained from the top-down solid state process. For both preparation methods, BM creates new non-radiative energy loss paths, possibly due to the formation of surface defects, which leads to a significant decrease in the PL intensity, a shorter PL lifetime, and weaker PersL performance.

To conclude, on one hand, for applications where particle size is not critical, such as anti-counterfeiting and lighting, ZGSO:0.5%Cr^3+^ prepared using the SS method shows more effective persistent luminescence properties than those prepared using the HT method, and is therefore the optimal choice. On the other hand, when nanoparticles are required, such as in bioimaging and theranostics [58,59,60], ZGSO:0.5%Cr^3+^ HT and BM NPs are indeed more interesting, presenting higher luminescent intensity and longer PersL duration compared to ZGSO:0.5%Cr^3+^ SS and BM NPs. This is observed in both dry powder and suspension.

## Data Availability

The data presented in this study are available on request from the corresponding author.

## Supplementary Materials

The following supporting information can be downloaded at: https://www.mdpi.com/article/10.3390/nano13152175/s1, Figure S1. Lifetime decay profiles of Zn_1.33_Ga_1.335_Sn_0.33_O_4_:Cr^3+^ (ZGSO:0.5%Cr^3+^) phosphors prepared by (a) solid-state and (b) hydrothermal methods before BM, under the different wavelengths of the laser excitation. Figure S2. (a) Crystal structure of the ZnGa_2_O_4_ normal spinel. The blue spheres represent Zn, red spheres represent O, and yellow spheres represent Ga. (b) Polyhedral view of a complex spinel. Tetrahedral sites are expected to be filled by Zn^2+^ ions, the octahedral sites are filled by the remaining Zn^2+^ ions and all Sn^4+^ and Ga^3+^ ions.
