# Supplementary material for: Effect of the Elaboration Method on Structural and Optical Properties of Zn1.33Ga1.335Sn0.33O4:0.5%Cr3+ Persistent Luminescent Nanomaterials
Source: Nanomaterials (Basel). 2023 Jul 26;13(15):2175. doi: 10.3390/nano13152175 (PMC10421510; doi:10.3390/nano13152175)
Supplement: Supplementary file 1 [file nanomaterials-13-02175-s001.zip › nanomaterials-2504922-supplementary.pdf]

Supplementary information

Effect of the Elaboration Method on Structural and Optical Properties of $Zn_{1.33}Ga_{1.335}Sn_{0.33}O_4$:0.5%$Cr^{3+}$ Persistent Luminescent Nanomaterialss

Guanyu Cai [1,2,†], Luidgi Giordano [1,†], Cyrille Richard [2,*] and Bruno Viana [1,*]

1 Chimie ParisTech, CNRS, Institut de Recherche de Chimie Paris (IRCP), Université PSL, 75005 Paris, France; guanyu.cai@chimieparistech.psl.eu (G.C.); luidgi.giordano@chimieparistech.psl.eu (L.G.)

2 Université Paris Cité, CNRS, INSERM, Unité de Technologies Chimiques et Biologiques pour la Santé (UTCBS), Faculté de Pharmacie, 75006 Paris, France

* Correspondence: cyrille.richard@u-paris.fr (C.R.); bruno.viana@chimieparistech.psl.eu (B.V.)

† These authors contributed equally to this work.

Fig. S1. Lifetime decay profiles of $Zn_{1.33}Ga_{1.335}Sn_{0.33}O_4:Cr^{3+}$ (ZGSO:0.5%Cr^{3+}) phosphors prepared by (a) solid-state and (b) hydrothermal methods before BM, under the different wavelengths of the laser excitation.

Figure S2. (a) Crystal structure of the $ZnGa_2O_4$ normal spinel. The blue spheres represent Zn, red spheres represent O, and yellow spheres represent Ga. (b) Polyhedral view of a complex spinel. Tetrahedral sites are expected to be filled by Zn^{2+} ions, the octahedral sites are filled by the remaining Zn^{2+} ions and all Sn^{4+} and Ga^{3+} ions.

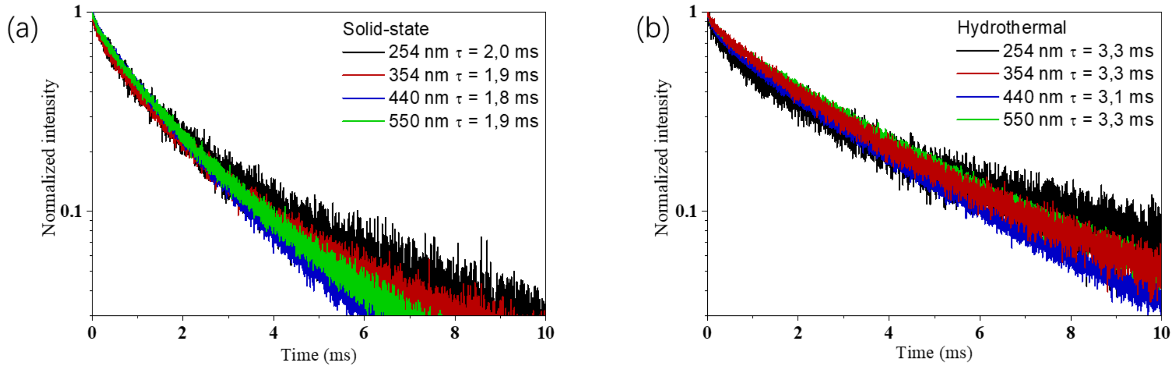

Figure S1. Lifetime decay profiles of $Zn_{1.33}Ga_{1.335}Sn_{0.33}O_4:Cr^{3+}$ (ZGSO:0.5%Cr^{3+}) phosphors prepared by (a) solid-state and (b) hydrothermal methods before BM, under the different wavelengths of the laser excitation.

Lifetime usually plays an important role in the evaluation of the luminescent property of optical materials. In the present study, ~ 3.1 ms and ~ 1.9 ms for the Cr^{3+}lifetime values are respectively measured for samples prepared by hydrothermal and solid-state methods. For all tested excitation wavelengths, there lifetime values do not strongly vary. The results are collected in Fig. S1. See text for the explanation of the difference between the two NPs family.

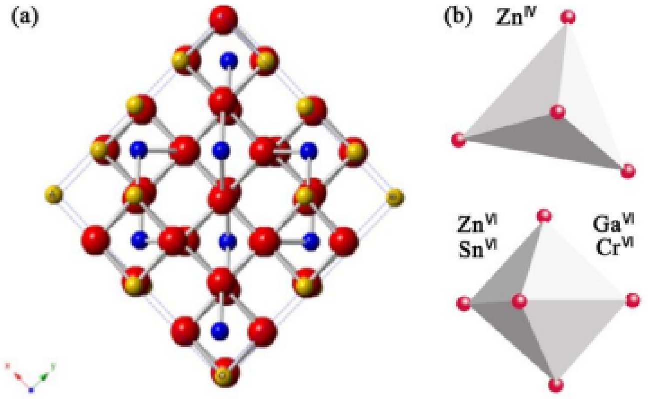

Figure S2. (a) Crystal structure of the $ZnGa_2O_4$ normal spinel. The blue spheres represent Zn, red spheres represent O, and yellow spheres represent Ga. (b) Polyhedral view of a complex spinel. Tetrahedral sites are expected to be filled by Zn^{2+} ions, the octahedral sites are filled by the remaining Zn^{2+} ions and all Sn^{4+} and Ga^{3+} ions.

ZGSO matrix possesses *normal spinel* (with a cationic substitution) and belongs to the cubic lattice system with two tetrahedral and four octahedral sites per formula unit. Ga^{3+} ions occupy the octahedral sites, while Zn^{2+} ions occupy two different positions: part of these Zn^{2+} ions occupy tetrahedral sites, and the rest occupy the octahedral sites (along with the Sn^{4+} ions). Thus, the ZGSO matrix is a cubic close-packed oxide with space group *Fd-3m*.
